# Long working hours and sickness absence—a fixed effects design

**DOI:** 10.1186/s12889-018-5473-y

**Published:** 2018-05-02

**Authors:** Vilde Hoff Bernstrøm

**Affiliations:** Work Research Institute, OsloMet – Oslo Metropolitan University, Oslo, Norway

**Keywords:** Sickness absence, Long working hours, Work hours, Fixed effects

## Abstract

**Background:**

While long working hours seem to lead to impaired health, several studies have also shown that long working hours are related to lower levels of sickness absence. Previous studies on the relationship between long working hours and sickness absence have compared those who work long hours to those who do not, looking only at between-individual correlations. Those results might therefore reflect relatively stable differences between employees who typically work long hours and employees who typically do not. The aim of the present study is to examine within-individual correlations between long working hours and sickness absence.

**Methods:**

Records from the Human Resources department in a large Norwegian hospital from 2012 to 2015 provided objective data on both working hours and sickness absence. Two analyses were performed: a prospective cohort analysis to replicate the results from previous between-individual analyses and a second analysis of within-individual correlations using a fixed effect design.

**Results:**

In line with existing research, both between-individual and within-individual analyses showed a negative relationship between long working hours (> 48 h/week) and short-term sickness absence (1–8 days) and no significant difference in incidence of long-term sickness absence (> 8 days).

**Conclusions:**

The results indicate that the negative relationship between long working hours and sickness absence is not due only to relatively stable individual differences between those who typically work long hours and those who do not. The results from both analyses therefore still contrast with previous research showing a negative relationship between long working hours and other health indicators.

## Background

Sickness absence represents a substantial cost for organizations and the state [1] and can have negative ramifications for the individual, including reduced well-being, alienation, and exit from the workforce [2–5]. As sickness absence is often also of particular interest as a readily measurable indicator of employee health, it is therefore important to understand the causes of sickness absence. The aim of the current paper is to investigate the relationship between long working hours and sickness absence.

Several studies have shown that long working hours are related to impaired health [6, 7]. Negative outcomes of working long hours include diabetes [8], depression and anxiety [9, 10], fatigue [11], mortality risk [12], increased risk of accidents [13], and coronary heart disease [14, 15]. Several of these ailments can, in turn, also be expected to increase employees’ risk of sickness absence. However, while long working hours have been found to be related to poorer health, long working hours have also been found to be related to reduced risk of sickness absence [16].

A recent systematic literature review of working hours and sickness absence identified 17 papers investigating the relationship between long working hours and sickness absence. Collectively, these found moderately strong support for a negative relationship between long working hours and sickness absence [16]. Of the 17 papers, five were prospective cohort studies, all of which found a significant negative relationship between long working hours and sickness absence [17–21]. None of the 17 identified studies used longitudinal data to investigate how individuals’ odds of sickness absence altered when their working hours altered (i.e., within-individual differences) [16]. The aim of the present paper is to look at these within-individual differences.

In particular, the results from the literature review [16] support the view that working more than 48–50 h per week is related to reduced levels of sickness absence. However, the relationship between sickness absence and working 40–48 h per week was less conclusive. The relationship between working hours and sickness absence also tended to be stronger for shorter spells of absence. Shorter absences are generally operationalized as self-certified absences or absences lasting up to three days. Two papers included more than one measure of sickness absence and found a significant relationship between long working hours and shorter spells of absence, but not between long working hours and longer spells [17, 19].

Several explanations have been advanced to account for the negative relationship between long working hours and sickness absence, which is the opposite of what would be expected in light of the relationship between long working hours and other health indicators. Several researchers have suggested that the negative relationship between long working hours and sickness absence is caused by a selection effect, often referred to as the “healthy worker effect,” where healthier employees choose to work longer hours [19, 22, 23]. It has also been suggested that this negative relationship is due to differences in work characteristics or conditions. For example, employees who work long hours may have jobs that are healthier or more motivating, or may have more scope to attend while ill. Krantz and Lundberg [22] found that a higher percentage of employees in top-level positions work long hours. Finally, some authors have suggested that the negative relationship between working hours and sickness absence is caused by differences in attendance motivation, arguing that high attendance motivation will induce employees to work longer hours and to attend work while ill (i.e., presenteeism) [17, 24]. Attendance motivation may be internal (e.g., caused by job satisfaction) or external (e.g., due to job insecurity). The latter argument is supported by studies showing that long working hours are related to a higher degree of presenteeism [25, 26]. Differences in attendance motivation would also explain why long working hours is related primarily to short spells of sickness absence but not to long absences, as the latter are more likely to have unavoidable causes [17].

As noted above, all of the studies identified in the systematic literature review compared employees who worked long hours to those who did not [16]. One important question, then, is whether the negative relationship between long working hours and sickness absence is caused by relatively stable characteristics of the individual or by their job. Additionally, it is important to ask how the individual’s risk of sickness absence alters when their working hours are altered.

It is possible that employees who typically work longer hours are healthier, have healthier working conditions, and/or are generally more motivated to attend work, which would mean that their levels of sickness absence are lower on average. However, these employees might still experience strain as a result of working long hours, with a subsequent increase in sickness absence as compared to when working shorter hours.

To understand the relationship between working hours and sickness absence, it is necessary to test whether within- and between-individual effects are the same. Of particular relevance is how an individual employee’s risk of sickness absence can be expected to alter when their working hours increase. The consequences of long working hours are also highly relevant from a legal and policy perspective. In Norway, the law dictates that the normal working week should not exceed 40 h [27]. However, upon written agreement, the number of hours worked can be calculated as an average so that they do not exceed 40 h per week over a 52-week period, 48 h during an eight-week period, or 54 h during a period of seven days [27]. Similarly, the EU Working Time Directive specifies that weekly working hours must not exceed 48 h *on average* [28].

The aim of the current paper, then, is to test the within-individual correlation between long working hours and sickness absence, classifying that absence as short (1–8 days), long (9–183 days) or extra-long (> 183 days) and based on data from a large Norwegian hospital for the period 2012–2015. Using a fixed effects design, this study is the first to investigate within-individual correlation between long working hours and sickness absence. To assess whether any discrepancies between the present findings and previous studies are due to differences in sampling, data, or methods of analysis, a prospective cohort analysis was also performed on the same data.

## Method

### Participants

#### The hospital

The study utilized data for the period 2012–2015 from a large Norwegian hospital. Following a merger of three different hospitals in 2010, the hospital has more than 20,000 employees. In addition to providing health care, it is also a research center and teaching hospital. Geographically, the hospital is spread across multiple locations in the capital of Norway.

For the hospital’s employees, the number of hours constituting a full-time position (i.e., not counting overtime) varies from 33 to 40 h per week, depending on the position. In most cases, a full-time contract means working 35.5 to 37.5 h per week, but a majority of physicians are contracted to work 38 to 40 h per week. Among ambulance personnel, a full-time position commonly involves less than 35 h per week.

In this hospital, as in Norway more generally, sickness absence attracts full pay from day one, for up to one year. The first 16 days are paid by the hospital, and subsequent days are reimbursed by the state. A medical certificate is needed for absences of nine days or more.

#### The employees

**Fig. 1:**
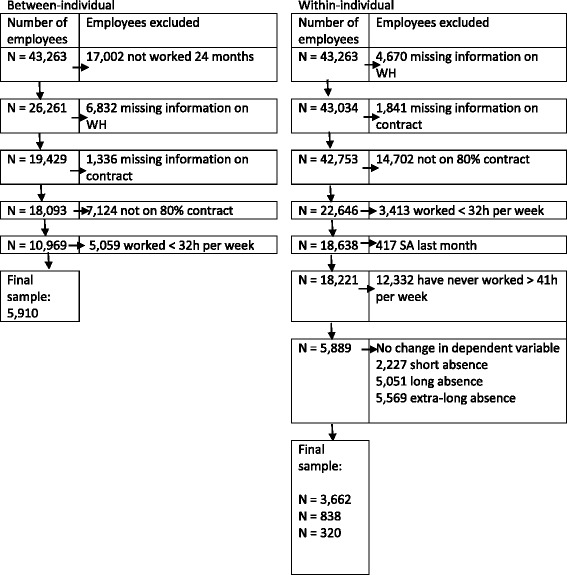
Sample selection

Hospital records for the study period contained 43,263 unique employee ID numbers. Selection criteria for inclusion in the final samples (as described below) are summarized in Fig. 1. Selection criteria for the between-individual analyses were by year, and by month for the within-individual analyses.

The between-individual analyses examined the relationship between working hours over one year and sickness absence during the following year. Employees who had not worked for at least two full calendar years without breaks in their contracts were therefore excluded from the between-individual analyses. For those employed for more than two years, data were included for the last two complete calendar years.

Any employees with missing information about their working hours or contract during the study period were excluded from both analyses. These were mainly employees who had not logged hours because of weak employment relations (e.g., temps called in on a day-to-day basis) and those who only worked at the hospital in the first years of the study period. Several departments did not start using the system for registering working hours (which was used to collect data for the present study) until 2013. For some employees, contract information may have been incomplete because of imprecise start and end dates. (To preserve anonymity, the hospital transformed start and end dates to quarters before sharing the data). In a few cases, there were errors in the registry.

To make the reference group data easier to interpret, both analyses were confined to full-time employees by restricting the sample to those with at least 80% cumulative employment contracts and who had worked at least 32 h per week on average. By using both logged working hours and contract as selection criteria, the sample excluded those who were on full-time contracts but were absent or on leave (e.g., parental leave) for a significant part of the year. The between-individual analyses also excluded employees who had changed contracts during the year and worked only 80% for parts of the year, as well as employees who had not logged hours for the entire year. For the within-individual analyses, this restriction also excluded months in which employees had worked limited hours because of vacation or other time-off periods.

Finally, a few additional selection criteria were used for the within-individual analyses. To avoid measuring absence in both the dependent and independent variable, the analysis excluded the month following any month in which an employee was absent. Otherwise, because sickness absence means working fewer hours, the correlation between working hours one month and the odds of sickness absence the next month would be influenced by the correlation between sickness absence one month and sickness absence the next. Additionally, because this analysis considered only variations within individuals, only those with variations in the dependent and independent variables could provide information of interest. On that basis, the analysis was restricted to employees who had worked for more than 41 h a week for at least one month. Furthermore, each analysis automatically restricted the sample to those varying on the dependent variable. The final sample (N) was therefore smaller for the analysis of longer spells of absence, as fewer employees exhibited variation in that outcome variable.

### Data

The data comprised three merged registries from separate hospitals for the years 2012 to 2015, based on scrambled employee ID numbers. The use of Human Resource department records made it possible to track changes in working hours and sickness absence over years for the same individuals, using objective data with a high level of accuracy.

#### Demographic information

The first registry contained contract and demographic information, including salary, age, gender, country of origin, position, and nature of contract (i.e., temporary or permanent). Country of origin was categorized as Norwegian, other Nordic countries, other Western countries, and non-Western countries. Work positions were categorized as physician, nurse, other patient-oriented position, administration/management, kitchen/cleaning/orderly, other operations (e.g., IT), and other.

As some employees had more than one employment contract, this information was summarized when possible (e.g., total salary), based on the contract with the highest percentage of a full-time equivalent for variables where it was impossible to summarize in this way (e.g., temporary or permanent position). A dummy variable was used to indicate that the employee held more than one contract.

#### Sickness absence

The second registry contained information about absences. Each spell of absence was registered as a start and end date and absence percentage (i.e., if an employee was 100% absent or partially absent). All spells of absence were included in the analyses, regardless of the percentage. Spells of absence were merged where one began the day after another ended. The data were aggregated to indicate whether an employee began a short spell of absence (1–8 days), a long spell of absence (9–183 days), or an extra-long spell of absence.

(> 183 days) in the given period. Distinguishing between short and long spells of absence was important because shorter spells are also influenced, to a greater extent than longer spells, by factors other than health [29, 30]. Shorter absences were operationalized as 1–8 days because a medical certificate is necessary only for absences lasting nine days or more. Extra-long spells of absence of more than 183 days were included to reflect the expectation that long working hours are also likely to be related to relatively severe health outcomes.

#### Working hours

The third registry contained information about the number of hours spent working and recorded each shift worked by each employee, along with start time, end time, organizational unit, and type of shift. As these records are used to calculate salaries, they are likely to be relatively accurate. Approximately 8% of employee-months included on-call shifts, and approximately 5% of employee-months included shifts registered as combining active and on-call work. To make the analysis less comprehensive, the combination shifts were recorded in terms of active and on-call hours, based on a registry indicator of equivalent active hours—in other words, a seven-hour-long combination shift registered as equivalent to four active hours was coded as four active hours and three on-call hours.

The data were aggregated to include the number of hours working active shifts and the number of hours on call during each month and year. For the between-individual analyses, average weekly working hours were calculated by dividing the number of hours spent working during the year by 230 (because a full-time employee in Norway is expected to work approximately 230 days a year) and then multiplying it by five (the number of working days per week). For the second analysis (within-individual), average weekly working hours were calculated by dividing the number of working hours in the month by the number of calendar days in that month and multiplying by seven.

This study focused on active shifts, categorizing active working hours (in accordance with the previous literature) as averaging 32–40, 41–48, or >  48 h a week. The reference group was 32–40 h. On-call shifts were included as a dummy control variable, indicating whether the employee also worked on call in the given month and year.

### Statistical analyses

For the first (between-individual) analyses, the data were analyzed using random effects, with a random intercept per work unit, controlling for the fact that work tasks and environments differ between units, and that employees are nested within their respective units.

For the second (within-individual) analyses, the data were analyzed using fixed effects, looking only at variation within individual employees. In this way, the fixed effect analysis controlled for all stable differences between individuals.

## Results

Employee demographic data are presented in Table 1, which shows that the hospital employs a diverse workforce that includes physicians, nurses, accountants, PR personnel, engineers, and cleaning staff, as well as workers in other fields.

**Table 1:** Demographic variables

	Prospective cohort	Fixed effects (all)	Fixed effects (worked > 41 h)	Fixed effects (worked > 48 h)
N	5910	18,221	5889	1631
Long working hours (> 48 h)	1%	9%	28%	100%
Semi-long hours (41–48 h)	6%	31%	96%	86%
Short sickness absence	73%	64%	64%	52%
Long sickness absence	23%	14%	14%	13%
Extra-long sickness absence	12%	6%	6%	5%
Age	45 (SD 12)	42 (SD 12)	43 (SD 12)	44 (SD 11)
Salary	506,685 (SD 160094)	649,470 (SD 369401)	746,052 (SD 438982)	887,558 (SD 479854)
Female	67%	71%	58%	43%
Temporary contract	16%	32%	38%	45%
Multiple jobholders	3%	5%	5%	6%
Norwegian	92%	92%	92%	91%
Other Nordic	3%	4%	4%	4%
Other western	2%	2%	2%	3%
Non Western	2%	2%	2%	2%
Physician	11%	16%	37%	67%
Nurse	30%	32%	27%	12%
Other patient-related	20%	22%	14%	10%
Administration/management	23%	17%	10%	6%
Kitchen/cleaning/orderly	6%	4%	5%	0%
Other operations	6%	4%	4%	2%
Other	6%	5%	3%	2%

Demographics for fixed effects shows percentage of employees with at least one instance of the given variable. N refers to all employees eligible to be included; for the fixed effects analyses, only those employees with variation in their dependent variable were included in the final analyses

The different employee groups exhibit clear differences in their propensity to work long hours. While physicians make up only 16% of the total sample, 67% of those employees who worked 48 h a week (on average during a month) are physicians. Similarly, while 71% of employees are female, only 43% of the employees who worked 48 h a week are female.

The results of the prospective cohort analysis (Table 2) show significantly lower odds of short-term (1–8 days) sickness absence for employees working long hours (41–48 h and >  48 h) but no difference in long-term sickness absence (> 8 days).

**Table 2:** Working hours and sickness absence—Prospective cohort analysis

	Short sickness absence (1–8 days)		Long sickness absence (9–183 days)		Extra-long sickness absence (> 183 days)	
	OR	95% CI	OR	95% CI	OR	95% CI
Long working hours (> 48 h per week)	0.40	(0.22–0.73) ***	0.62	(0.26–1.48) ns	0.40	(0.10–1.69) ns
Semi-long working hours (41–48 h per week)	0.56	(0.43–0.73) ***	0.83	(0.60–1.13) ns	0.90	(0.60–1.35) ns
Salary	1.00	(1.00–1.00) ***	1.00	(1.00–1.00) ***	1.00	(1.00–1.00) ns
Temporary contract	0.70	(0.57–0.86) ***	0.74	(0.60–0.92) ***	0.72	(0.55–0.94) *
Multiple job holder	0.94	(0.66–1.35) ns	0.96	(0.67–1.37) ns	0.89	(0.56–1.40) ns
Female	1.56	(1.35–1.81) ***	1.62	(1.39–1.88) ***	1.81	(1.48–2.20) ***
Age	0.98	(0.98–0.99) ***	1.00	(0.99–1.01) ns	0.99	(0.98–1.00) *
On-call shifts	0.95	(0.75–1.20) ns	0.84	(0.65–1.08) ns	0.85	(0.62–1.17) ns
Country of origin						
Norwegian (Control)						
Nordic immigrant	1.51	(1.03–2.23) *	0.77	(0.53–1.12) ns	0.76	(0.47–1.23) ns
Other Western immigrants	1.07	(0.69–1.68) ns	1.14	(0.73–1.80) ns	0.87	(0.46–1.65) ns
Non-Western immigrants	0.92	(0.58–1.44) ns	0.95	(0.61–1.48) ns	0.63	(0.33–1.23) ns
Job						
Nurse (control)						
Physician	0.97	(0.72–1.30) ns	1.21	(0.87–1.66) ns	1.03	(0.69–1.54) ns
Patient-oriented other	0.95	(0.76–1.18) ns	1.24	(1.04–1.49) *	1.24	(1.00–1.54) *
Administration/management	0.67	(0.55–0.82) ***	0.91	(0.76–1.10) ns	0.95	(0.76–1.20) ns
Other operations	0.66	(0.48–0.90) ***	1.39	(1.05–1.84) *	1.24	(0.87–1.77) ns
Kitchen/cleaning/orderly	1.35	(0.892.05) ***	1.36	(1.02–1.83) *	0.78	(0.52–1.18) ns
Other job	0.22	(0.16–0.30) ns	0.63	(0.44–0.90) **	0.78	(0.51–1.18) ns
Cons	26.27		0.43		0.21	
Variance (random-part)		SE		SE		SE
Level 1: Employee	π2/3		π2/3		π2/3	
Level 2: Work unit	0.29	0.00	0.05	0.01	0.00	0.00

Logistic regression. Random effects analyses; random intercept for work unit

N employees: 5910; N departments: 986 (Full-time employees only)

The random part of the table presents the estimated variance explained at each level

* *p* > 0.05; ** *p* > 0.01; *** *p* > 0.001

The fixed effects analyses (Table 3) showed significantly lower odds of short sickness absence for individual employees after working long hours, with no difference for semi-long working hours (41–48) or for longer sickness absences.

**Table 3:** Working hours and sickness absence—Fixed effects analysis

	Short sickness absence (1–8 days)		Long sickness absence (9–183 days)		Extra-long sickness absence (> 183 days)	
	OR	95% CI	OR	95% CI	OR	95% CI
Long Working hours (> 48 h per week)	0.77	(0.66–0.90) **	0.88	(0.62–1.26) ns	0.81	(0.45–1.45) ns
Semi-long working hours (40–48 h per week)	0.98	(0.92–1.05) ns	0.93	(0.79–1.10) ns	0.92	(0.69–1.22) ns
Salary	1.00	(1.00–1.00) ns	1.00	(1.00–1.00) ns	1.00	(1.00–1.00) ns
Temporary contract	0.67	(0.58–0.78) ***	0.46	(0.29–0.71) **	0.61	(0.31–1.19) ns
Multiple job holder	0.77	(0.55–1.08) ns	1.54	(0.58–4.07) ns	2.09	(0.52–8.47) ns
On-call shifts	0.94	(0.80–1.11) ns	0.85	(0.56–1.31) ns	1.02	(0.54–1.93) ns
January to March	1.11	(1.03–1.19) **	01.38	(1.14–1.67) **	1.55	(1.12–2.14) **
April to June	0.74	(0.69–0.80) ***	0.97	(0.79–1.20) ns	1.19	(0.84–1.68) ns
July to September	0.57	(0.52–0.61) ***	0.92	(0.76–1.13) ns	0.87	(0.61–1.24) ns

Logistic regression. Fixed effects analyses; employee-months nested within employees

N short absence: 3662 (40,513 observations); employees followed 2–32 months (11 on average) N long absence: 838 (9434); employees followed 2–27 months (11 on average) N extra-long absence: 320 (3629); employees followed 2–26 months (11 on average)

** p > 0.01;*** p > 0.001

Additional analyses (not shown) tested the robustness of the results of fixed effects analysis. Analyses using working hours over the previous six months as the independent variable did not yield substantially different results. Similarly, a single analysis of all medically certified absences (> 8 days) (to strengthen the analysis by increasing the number of employees with at least one spell of absence) did not yield substantially different results. Again, analyzing only ≤3 days of absence—the spell returning the most consistent findings in previous studies—yielded similar findings.

## Discussion

As expected, and in line with the previous literature, the prospective cohort analyses showed significantly lower incidence of short-term sickness absence (1–8 days) for employees working long hours (both 41–48 h and >  48 h). As there was no significant relationship between working long hours and odds of long or extra-long sickness absences, the results also support previous findings that working long hours relates mainly to lower odds of short sickness absences and not necessarily to longer absences [17, 19, 21]. In general, the findings from the prospective cohort analysis are very similar to the results of previous cross-sectional and prospective cohort analyses.

Notably, the fixed effects analysis did also indicate a significant relationship between working long hours (> 48 h) and short-term sickness absence. This is the first study to test this within-individual relationship.

### Practical and theoretical implications of the findings

The current results have three especially important implications, the first of which relates to how we interpret the negative relationship between long working hours and sickness absence. While the between-individual analyses’ support for relatively stable individual differences might partly explain the lower absence rate among employees who work long hours, the relationship does not seem entirely due to stable differences between individuals. The within-individual relationship may indicate that employees regulate how much they work in accordance with fluctuations in their own health—that is, they work more when they feel fit. Attendance motivation may also fluctuate according to work demands, as employees may be more reluctant to call in sick during periods of high work pressure because of an increased sense of being needed or increased guilt about leaving already pressured colleagues to do more work.

Second, in line with two previous studies, the present results only showed a significant relationship between long working hours and short-term sickness absence [17, 19], supporting the view that lower sickness absence rates among those working long hours are likely to be caused by those factors that are most relevant for shorter absences. If employees who work longer hours were significantly healthier, we would also expect to find a relationship between long working hours and long-term sickness absence. Attendance motivation and more scope to attend work while ill might be more relevant explanations when only short-term absence is lower.

Third and notably, these results do not align with previous research showing that increased health problems are a consequence of long working hours [6, 7]. We might expect that, after controlling for stable differences between employees and their work, the results would show increased absence following periods of long working hours. While employees who work longer hours typically also have lower absence rates, they may nevertheless experience strain as a result of working long hours, with increased absence as compared to when working shorter hours. However, the results do not support any such relationship. Arguably, one explanation for the lack of a positive correlation in the fixed effects study between long working hours and sickness absence is that some health problems may take time to manifest. However, looking at working hours over the previous six months did not alter these results substantially. The lack of a positive relationship between working hours and sickness absence might also be an effect of the sample. Some studies have reported that the negative health consequences of long working hours are found mainly among blue-collar workers [8, 12]; in the current sample, it is mainly white-collar employees who work long hours, especially physicians. Future studies should further explore why no positive correlation was found here between long working hours and sickness absence.

As mentioned earlier, current legal policies allow employers to calculate average weekly working hours over an extended period, so allowing for shorter periods of long working hours [27, 28]. The results indicate no negative ramifications of such policies in relation to sickness absence.

### Limitations and strengths

Some of the study’s limitations and strengths are worth commenting on. The case hospital offered a unique opportunity to use objective longitudinal records of working hours, measuring both dependent and independent variables with high precision over 4 years. Using a fixed effects design, this study is the first to investigate within-individual correlation between long working hours and sickness absence.

Although this was a large heterogeneous sample, the employees who work long hours are mainly white-collar groups. This limits the generalizability of the findings, and their application to populations of blue-collar workers who work long hours requires further investigation. However, the similarities between the results of previous analyses and the first cohort analyses in the present study tend to support the study’s generalizability.

The final samples, consisting only of full-time employees who log at least 32 h a week on average, excludes a large number of employees who did not meet all the inclusion criteria. These exclusions are important for the interoperation of the results. The relationship between work hours and sickness absence is unlikely to be linear [16], and including part-time employees in the control group could have influenced the results. Furthermore, because both analyses are longitudinal (prospective cohort and fixed effect) the sample is restricted to employees who have worked at least two consecutive periods (2 years for the prospective cohort and 2 months for the fixed effects analyses). Some employees in the hospital sector work on relatively short-term contracts and they might differ from permanent employees both in terms of pressure to work long hours and pressure to attend work while ill.

Finally, the use of objective records provides no information about employees’ motives and experiences. Additional information, such as why employees work long hours, may yield more nuanced results.

## Conclusions

While long working hours seem to lead to impaired health, several studies have also shown that long working hours are related to lower levels of sickness absence. In line with previous studies of sickness absence, the present results showed significant between-individual differences indicating that employees who work longer hours have lower absence rates. One novel contribution of the current study was that it also investigated within-individual differences in relation to long working hours and sickness absence and how employees’ odds of sickness absence change when their working hours change. The results show a moderate reduction in the odds of short-term sickness absence after months of long working hours. These results indicate that the negative relationship between long working hours and short-term sickness absence is not only a consequence of stable differences between those who typically work long hours and those who typically do not. One possible explanation is that employees’ attendance motivation increases when work pressure is high, with an accompanying decrease in short-term sickness absence. These findings conflict with earlier studies reporting a relationship between long working hours and reduced health, and future studies should explore this contradiction.

Current legislation commonly emphasizes average working hours over an extended period, allowing for shorter periods of long working hours. The current study found no negative effect of long working hours on sickness absence in the hospital sector.

## References

[1] Nicholson S, Pauly MV, Polsky D, Sharda C, Szrek H, Berger ML (2006). Measuring the effects of work loss on productivity with team production. Health Econ.

[2] Floderus B, Goransson S, Alexanderson K, Aronsson G (2005). Self-estimated life situation in patients on long-term sick leave. J Rehabil Med.

[3] Vingard E, Alexanderson K, Norlund A (2004). Chapter 9. Consequences of being on sick leave. Scand J Public Health.

[4] Sieurin L, Josephson M, Vingard E (2009). Positive and negative consequences of sick leave for the individual, with special focus on part-time sick leave. Scandinavian Journal of Public Health.

[5] Markussen S (2012). The individual cost of sick leave. J Popul Econ.

[6] Lie J-AS, Arneberg L, Goffeng LO, Graveseth HM, Lie A, Ljoså GH, Matre D (2014). Arbeidstid og helse. Oppdatering av en systematisk litteraturstudie. In*.*, vol. 15.

[7] Van der Hulst M. Long workhours and health. Scand J Work Environ Health. 2003:171–88. 10.5271/sjweh.720 12828387

[8] Kivimäki M, Virtanen M, Kawachi I, Nyberg ST, Alfredsson L, Batty GD, Bjorner JB, Borritz M, Brunner EJ, Burr H (2015). Long working hours, socioeconomic status, and the risk of incident type 2 diabetes: a meta-analysis of published and unpublished data from 222 120 individuals. Lancet Diabetes Endocrinol.

[9] Virtanen M, Ferrie JE, Singh-Manoux A, Shipley MJ, Stansfeld SA, Marmot MG, Ahola K, Vahtera J, Kivimäki M (2011). Long working hours and symptoms of anxiety and depression: a 5-year follow-up of the Whitehall II study. Psychol Med.

[10] Virtanen M, Stansfeld SA, Fuhrer R, Ferrie JE, Kivimäki M (2012). Overtime work as a predictor of major depressive episode: a 5-year follow-up of the Whitehall II study. PLoS One.

[11] Tucker P, Brown M, Dahlgren A, Davies G, Ebden P, Folkard S, Hutchings H, Åkerstedt T. The impact of junior doctors' worktime arrangements on their fatigue and well-being. Scand J Work Environ Health. 2010:458–65. 10.5271/sjweh.2985 20414629

[12] O’reilly D, Rosato M (2013). Worked to death? A census-based longitudinal study of the relationship between the numbers of hours spent working and mortality risk. Int J Epidemiol.

[13] Nakata A (2011). Effects of long work hours and poor sleep characteristics on workplace injury among full-time male employees of small-and medium-scale businesses. J Sleep Res.

[14] Virtanen M, Ferrie JE, Singh-Manoux A, Shipley MJ, Vahtera J, Marmot MG, Kivimäki M (2010). Overtime work and incident coronary heart disease: the Whitehall II prospective cohort study. Eur Heart J.

[15] Holtermann A, Mortensen OS, Burr H, Søgaard K, Gyntelberg F, Suadicani P (2010). Long work hours and physical fitness: 30-year risk of ischaemic heart disease and all-cause mortality among middle-aged Caucasian men. Heart.

[16] Bernstrøm VH, Houkes I (2018). A systematic literature review of the relationship between work hours and sickness absence. Work & Stress.

[17] Ala-Mursula L, Vahtera J, Kouvonen A, Vaananen A, Linna A, Pentti J, Kivimaki M (2006). Long hours in paid and domestic work and subsequent sickness absence: does control over daily working hours matter?. Occup Environ Med.

[18] Hansen ML, Thulstrup AM, Juhl M, Kristensen JK, Ramlau-Hansen CH (2015). Occupational exposures and sick leave during pregnancy: results from a Danish cohort study. Scand J Work Environ Health.

[19] Laaksonen M, Pitkaniemi J, Rahkonen O, Lahelma E (2010). Work arrangements, physical working conditions, and psychosocial working conditions as risk factors for sickness absence: Bayesian analysis of prospective data. Ann Epidemiol.

[20] Magee C, Stefanic N, Caputi P, Iverson D (2011). Occupational factors and sick leave in Australian employees. J Occup Environ Med.

[21] Magee CA, Caputi P, Lee JK (2016). Distinct longitudinal patterns of absenteeism and their antecedents in full-time Australian employees. J Occup Health Psychol.

[22] Krantz G, Lundberg U (2006). Workload, work stress, and sickness absence in Swedish male and female white-collar employees. Scandinavian Journal Of Public Health.

[23] Niedhammer I, Chastang JF, Sultan-Taieb H, Vermeylen G, Parent-Thirion A (2013). Psychosocial work factors and sickness absence in 31 countries in Europe. Eur J Pub Health.

[24] Lesuffleur T, Chastang J-F, Sandret N, Niedhammer I (2014). Psychosocial factors at work and sickness absence: results from the French national SUMER survey. Am J Ind Med.

[25] Böckerman P, Laukkanen E (2010). What makes you work while you are sick? Evidence from a survey of workers. Eur J Pub Health.

[26] Hansen CD, Andersen JH (2008). Going ill to work–what personal circumstances, attitudes and work-related factors are associated with sickness presenteeism?. Soc Sci Med.

[27] Arbeidsmiljøloven: Lov om arbeidsmiljø, arbeidstid og stillingsvern mv. (arbeidsmiljøloven). [act relating to working environment, working hours and employment protection, etc. (working environment act)] § 10. In*.*; 2007.

[28] Working Conditions - Working Time Directive [http://ec.europa.eu/social/main.jsp?catId=706&langId=en&intPageId=205 ].

[29] Kivimäki M, Head J, Ferrie JE, Shipley MJ, Vahtera J, Marmot MG: Sickness absence as a global measure of health: evidence from mortality in the Whitehall II prospective cohort study. Br Med J 2003, 327(7411):364–368. 10.1136/bmj.327.7411.364 PMC175810 12919985

[30] Marmot M, Feeney A, Shipley M, North F, Syme SL (1995). Sickness absence as a measure of health-status and functioning — from the UK Whitehall-II study. J Epidemiol Community Health.

